# Dose-dependent stress responses of the aquatic moss Taxiphyllum barbieri under acute X-ray exposure: implications for environmental risk assessment

**DOI:** 10.1007/s11356-026-38065-4

**Published:** 2026-07-16

**Authors:** Chiara Amitrano, Veronica De Micco, Gian Pietro Di Sansebastiano, Mariagabriella Pugliese, Cecilia Arrichiello, Paolo Muto, Stefania De Pascale, Carmen Arena

**Affiliations:** 1 Department of Agricultural Sciences, University of Naples Federico II, Portici (Naples), Italy; 2 Department of Biological and Environmental Sciences and Technologies (Di.S.Te.B.A.), University of Salento, Lecce, Italy; 3 Department of Physics Ettore Pancini, University of Naples Federico II, Naples, Italy; 4 Radiotherapy Unit, Istituto Nazionale Tumori—IRCCS—Fondazione G. Pascale, Naples, Italy; 5 Department of Biology, University of Naples Federico II, Naples, Italy

**Keywords:** Aquatic bryophytes, Biomonitoring, Ecotoxicology, Environmental monitoring, Hormesis, Ionizing radiation, Oxidative stress

## Abstract

Freshwater ecosystems may experience acute oxidative stress following accidental or localized release of ionizing radiation. In these environments, the responses of aquatic bryophytes remain poorly understood. We investigated dose-dependent physiological, biochemical, and structural responses of the aquatic moss *Taxiphyllum barbieri* after acute X-ray exposure (1, 10, and 30 Gy), aiming to investigate dose-dependent physiological responses and identify potential functional resilience thresholds. Photosynthetic performance, pigment composition, antioxidant activity, and structural traits were assessed over a 63-day recovery period using an integrated multilevel approach. Low-dose irradiation (1 Gy) stimulated net carbon assimilation, PSII efficiency, electron transport rate, and pigment content, indicating a low-dose stimulatory response consistent with hormesis. In contrast, higher doses (10–30 Gy) progressively constrained photosynthetic performance and pigment concentration, while inducing persistent alterations in phenolic metabolism and shoot architecture. Multivariate analyses showed marked early divergence from controls followed by partial physiological stabilization at 63 days, despite incomplete structural recovery. These findings highlight the capacity of *T. barbieri* to maintain key functional processes under acute oxidative stress and support the inclusion of ecophysiological endpoints in ecological risk assessment frameworks addressing episodic radiation exposure in freshwater systems.

**Graphical Abstract:**

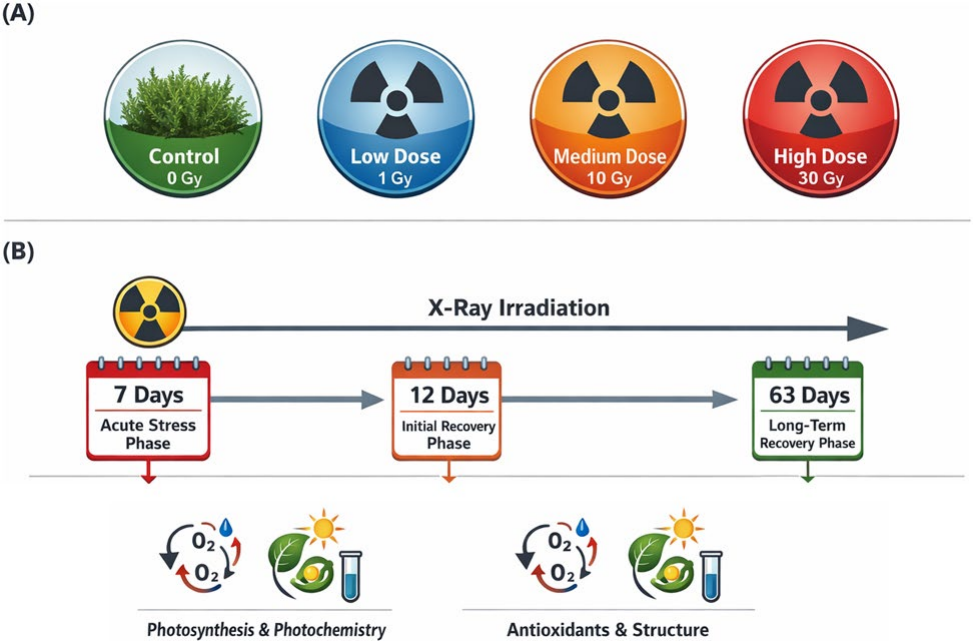

**Supplementary Information:**

The online version contains supplementary material available at 10.1007/s11356-026-38065-4.

## Introduction

Aquatic ecosystems are increasingly exposed to acute and chronic environmental stressors resulting from anthropogenic activities, including chemical pollution, thermal anomalies, and ionizing radiation associated with industrial, medical, and accidental releases or improper radioactive waste disposal. Among these stressors, ionizing radiation represents a particularly severe form of oxidative stress, capable of generating high amounts of reactive oxygen species (ROS), damaging cellular structures, and disrupting metabolic processes in aquatic organisms (Azzam et al. 2012; Einor et al. 2016). Large-scale nuclear accidents such as Chernobyl and Fukushima have demonstrated the persistence of radionuclides in freshwater ecosystems, where primary producers may experience both chronic and acute exposures (Møller and Mousseau 2006; Garnier-Laplace et al. 2015). Despite these documented scenarios, the ecological consequences of low-dose ionizing radiation have been extensively investigated in vascular plants and animals. In contrast, the responses of non-vascular aquatic primary producers to ionizing radiation remain poorly understood.

Aquatic plants play a significant role in freshwater ecosystems by contributing not only to primary production but also to water oxygenation, nutrient cycling, and contaminant removal. In recent decades, increasing attention has been devoted to the use of aquatic macrophytes and algae as bioindicators and biofilters in contaminated environments, due to their capacity to accumulate pollutants, acting as a functional buffer against stress propagation from organisms to ecosystem processes (Vymazal 2014; Rai 2018). Currently, most studies have focused on vascular plants or microalgae, while bryophytes, despite their well-documented stress tolerance, have received comparatively little attention in aquatic environmental research.

Bryophytes are non-vascular photoautotrophs characterized by simple morphology, high surface-area-to-volume ratios, and direct exchange with the surrounding environment. These traits confer a remarkable ability to tolerate abiotic stresses such as desiccation, heavy metal contamination, and oxidative stress, making bryophytes effective bioindicators of environmental quality (Proctor et al. 2007). In aquatic systems, mosses have been widely used to monitor metal pollution and nutrient enrichment, owing to their capacity to accumulate contaminants and reflect water chemistry over time (Samecka-Cymerman and Kempers 2000). Nevertheless, their functional responses, especially to extreme oxidative stressors—such as ionizing radiation—remain largely unexplored (Amitrano et al. 2025).

Ionizing radiation induces oxidative stress primarily through water radiolysis, leading to the formation of highly reactive radicals that target membranes, pigments, nucleic acids, and proteins (Amitrano et al. 2026; De Francesco et al. 2025). In photosynthetic organisms, radiation-induced ROS can impair chloroplast structure, disrupt electron transport, and reduce carbon assimilation efficiency. At the same time, low levels of oxidative stress may activate protective and compensatory mechanisms, including antioxidant metabolism, pigment remodeling, and structural plasticity. This biphasic response, commonly referred to as *hormesis*, has been documented in vascular plants exposed to various stressors, including radiation, metals, and UV exposure (Calabrese and Baldwin 2003; Calabrese and Mattson 2017). Whether similar hormetic patterns occur in aquatic bryophytes, and whether these responses translate into functional resilience, remains an open question. Here, functional resilience is defined as the capacity to stabilize key physiological processes following acute disturbance, rather than full structural or biochemical recovery (Gladstone-Gallagher et al. 2019), whereas physiological recovery refers more specifically to the partial or complete return of measured physiological traits toward pre-disturbance conditions.

Understanding the capacity of aquatic mosses to maintain physiological performance under acute stress is particularly relevant in the context of contaminated freshwater environments. Organisms capable of withstanding such stress while preserving key ecosystem functions—such as oxygen production and nutrient cycling—may contribute to the maintenance of ecosystem processes. However, the extent to which organism-level responses translate into ecosystem-level outcomes requires further investigation.

Although chronic low-dose exposure is common near contaminated sites, episodic radiation contamination can occur during accidental releases or mismanagement of radioactive wastes, making acute oxidative stress responses and physiological recovery environmentally relevant endpoints. Beyond terrestrial freshwater systems, analogous oxidative stress scenarios are encountered in space and planetary environments, where reduced atmospheric and magnetic shielding can expose biological systems to elevated ionizing radiation, making stress resilience a critical trait for biological sustainability in such extreme environments.

In this study, we investigated the dose-dependent physiological, morphological, and biochemical responses and recovery of the aquatic moss *T. barbieri* to acute X-ray irradiation. X-rays were used because they are the reference ionizing radiation in radiobiology for dose calibration and comparative biological effectiveness studies (Amitrano et al. 2018). Although real environmental contamination scenarios often involve chronic exposure and mixed radiation fields, X-rays provide a well-characterized and reproducible source of ionizing radiation that allows controlled investigation of radiation-induced oxidative stress mechanisms. Consequently, they are widely used in radiobiological studies as a reference model for evaluating dose-dependent biological responses (Azzam et al. 2012; Reisz et al. 2014). By combining real-time gas exchange monitoring, photosynthetic and photochemical analyses, pigment quantification, antioxidant profiling, and structural observations over a post-exposure recovery period, we aimed to assess the functional resilience of this species under oxidative stress. Specifically, we tested the hypothesis that *T. barbieri* exhibits a hormetic response to ionizing radiation, characterized by stimulation at low doses and inhibition at higher doses, also testing its capacity for physiological recovery over time. The selected dose range (1–30 Gy) was designed to encompass both low-dose exposures relevant for investigating early stress signaling to acute radiation-induced stress (1 Gy) and higher experimental doses aimed at defining physiological tolerance thresholds and resilience limits. Doses are therefore not intended to represent routine environmental conditions. Rather, they are commonly employed in plant radiobiology to characterize tolerance boundaries, identify transitions between adaptive and inhibitory responses, and investigate mechanisms of stress resilience (Esnault et al. 2010; Wi et al. 2007; Al-Enezi et al. 2012; ICRP 2014). Integrating sub-lethal functional endpoints into environmental risk assessment (ERA) is particularly relevant for primary producers, as early physiological impairments may cascade to ecosystem-level processes such as oxygen balance and carbon cycling.

Despite the widespread use of aquatic bryophytes as bioindicators of water quality, their eco-physiological and biochemical responses to acute oxidative stressors remain unexplored, and to our knowledge, no previous study has provided an integrated functional characterization of these mosses under ionizing radiation exposure. Recently, it was shown that the slow growth rate, usually considered a limiting factor in the use of these photosynthetic organisms, is not a limitation in vegetative lines of *T. barbieri* (Anglana et al. 2024), and applications are increasing (Del Piano et al. 2026). By providing a combined, multi-level assessment of radiation-induced stress responses in an aquatic bryophyte, this work would contribute to a broader understanding of plant resilience in contaminated environments and highlights the potential role of mosses as robust biological components in freshwater systems exposed to extreme abiotic stressors.

## Materials and methods

### Plant material and growth conditions

The aquatic moss *Taxiphyllum barbieri* (Mitt.) M. Fleisch. was obtained as semi-axenic biomass from a commercial in vitro culture (Green Greener srl, Policoro, Italy).

Mosses were cultivated in transparent polycarbonate containers filled with microfiltered water (electrical conductivity approximately 60 µS cm⁻^1^) without any solid substrate. Cultures were maintained in a growth chamber at 25 ± 2 °C under a 16/8 h light/dark photoperiod, with photosynthetic photon flux density (PPFD) set at 200 µmol photons m⁻^2^ s⁻^1^ using white LED lamps. Relative humidity in the chamber was maintained at approximately 80%. Water was renewed weekly to ensure chemical stability and prevent microbial contamination.

Before irradiation, moss samples were acclimated for 2 weeks under controlled environmental conditions to minimize handling and transfer-related variability. Immediately prior to exposure, excess surface moisture was gently blotted, and samples were adjusted to achieve uniform fresh weight across experimental units.

### X-ray irradiation treatments

X-ray irradiation was performed at the National Cancer Institute IRCCS Fondazione G. Pascale (Naples, Italy) using a medical linear accelerator (Elekta Versa HD with Agility MLC, Elekta AB, Sweden). Irradiation was delivered using 6 MV photon beams under a three-dimensional conformal radiotherapy (3D-CRT) geometry to ensure homogeneous dose distribution.

Moss samples were positioned between two Perspex slabs (2.5 cm and 5 cm thickness) to achieve accurate dosimetry. The irradiation field consisted of two opposing 20 × 20 cm^2^ beams, with a dose rate of 2 Gy min⁻^1^.

The following irradiation treatments were applied: non-irradiated control (CTRL), 1 Gy, 10 Gy, and 30 Gy.

Doses were selected to span from a low-stress signaling range to a high-stress impairment range. The dose selection was based on both radiobiological and ecological considerations. The lowest dose (1 Gy) was selected to evaluate potential adaptive responses associated with mild oxidative stress, whereas 10 and 30 Gy were included to progressively increase physiological pressure and identify tolerance limits. Although a denser dose gradient would provide a more detailed characterization of the dose–response relationship, the present study was conceived as an exploratory multi-endpoint assessment integrating physiological, biochemical, and structural responses across multiple recovery time points. Therefore, representative low-, intermediate-, and high-dose treatments were selected to capture broad response patterns and functional resilience rather than to establish a high-resolution dose–response model. In addition, irradiation experiments were conducted using a clinical linear accelerator within a hospital radiotherapy facility, with limitations in beamtime scheduling.

This logistical constraint further supported the selection of a restricted number of biologically informative dose levels while maintaining analytical consistency across all investigated endpoints.

Immediately after irradiation, moss samples were transferred back to the controlled growth conditions reported above. All irradiation treatments were performed on three independent biological replicates. For microscopy, fluorescence, and morphometric analyses, multiple measurements were obtained from each biological replicate (e.g., multiple fields of view or individual structures). To avoid pseudoreplication, measurements obtained within the same biological replicate were averaged prior to statistical analysis, and the biological replicate was considered the experimental unit throughout the study.

### Experimental design and continuous O₂ and CO₂ monitoring

**Fig. 1:**
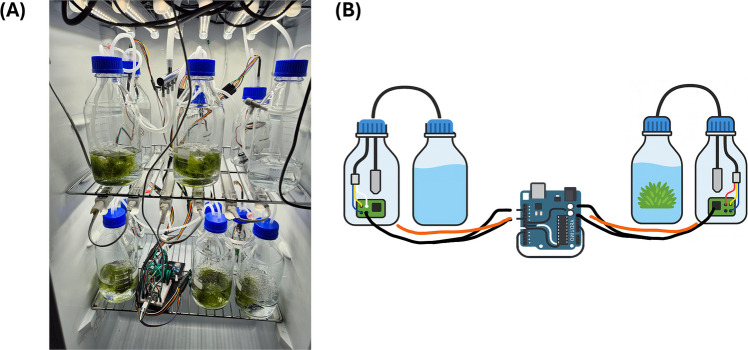
Real-time O₂ and CO₂ gas exchange monitoring in irradiated *T. barbieri*. **A** Experimental setup used for post-irradiation cultivation of *T. barbieri*. **B** Schematic representation of the closed-loop cultivation and gas-analysis system equipped with non-invasive O₂ and CO₂ sensors for continuous gas flux measurements

Following irradiation, moss samples were cultivated for up to 63 days to assess both short-term and long-term morpho-anatomical, physiological, and biochemical responses (Fig. 1A).

Each experimental unit consisted of an individual moss sample cultivated in a sealed transparent bottle filled with microfiltered water. A water-only control (blank) was included in all experiments to account for abiotic gas exchange and background signal (Fig. 1B).

To monitor photosynthetic gas exchange in real time, a closed-loop cultivation system was developed. Each moss-containing bottle was connected via airtight silicone tubing to a secondary gas-analysis chamber equipped with optical oxygen and carbon dioxide sensors (FireSting O₂ and CO₂, PyroScience GmbH, Germany).

The system allowed continuous, non-invasive measurement of O₂ and CO₂ concentrations in the headspace. Measurements were conducted over 24-h cycles under the controlled environmental conditions reported above.

Raw sensor data were corrected by subtracting values obtained from the water-only blank. Linear regressions of O₂ and CO₂ concentration changes over time were calculated using ordinary least squares. Gas fluxes were expressed as µmol h⁻^1^ g FW⁻^1^ and calculated using the ideal gas law:

$$n=\frac{PV}{RT}$$

where *n* is the amount of substance (mol), *P* is pressure (Pa), *V* is volume (m^3^), *R* is the gas constant, and *T* is temperature (K).

All subsequent measurements were performed at 7, 12, and 63 days after irradiation (DAI), depending on the parameter analyzed.

### Gas-exchange and chlorophyll fluorescence measurements

Net photosynthetic rate was measured using a portable infrared gas analyzer (LCPro +, ADC BioScientific Ltd., UK) equipped with a versatile chamber suitable for small aquatic plant samples.

Measurements were conducted at 12 and 63 DAI under controlled chamber conditions: temperature 25 ± 2 °C, CO₂ concentration approximately 400 ppm, relative humidity 50–60%, and PPFD set at 500 µmol photons m⁻^2^ s⁻^1^. Samples were allowed to acclimate in the chamber for 15–30 min prior to data acquisition to ensure a steady-state condition. Net CO₂ assimilation rates were expressed as µmol CO₂ m⁻^2^ s⁻^1^.

Chlorophyll fluorescence was assessed, at 12 and 63 DAI, using a pulse-amplitude modulated fluorometer (Junior-PAM, Walz GmbH, Effeltrich, Germany).

The optical fiber (1 mm diameter) was positioned at 0.5 mm from the sample surface and inclined at 45°. The basal fluorescence (F_0_) was recorded using a weak blue light beam (1–2 μmol photons m^−2^ s^−1^), while maximal fluorescence in the dark-adapted state (F_m_) was induced by a 0.8-s saturating pulse of 7000 μmol photons m^−2^ s^−1^. Maximum PSII photochemical efficiency (F_v_/F_m_) was calculated as (F_m_-F_0_)/F_m_ (Kitajima and Butler 1975) and used as indicator of photosystem health.

The measurements in the light were conducted at growth irradiance (200 µmol photons m⁻^2^ s⁻^1^).

Briefly, the effective quantum yield of PSII electron transport (Φ_PSII_) was calculated following the equation reported in Genty et al. (1989); the electron transport rate (ETR) was calculated according to Schreiber (2004), while non-photochemical quenching (qN) was measured as reported in Maxwell and Johnson (2000).

### Microscopic morphological and autofluorescence analyses

For microscopic analyses, samples were fixed in FAA solution (5% formaldehyde/5% acetic acid/90% ethanol 50%, v/v/v) and observed under a light microscope (Olympus BX53, Olympus Corporation, Japan) equipped with a digital camera (Olympus DP23).

To monitor moss growth, the ramification density (number of branches per mm^2^) and length (mm) were quantified using ImageJ software (National Institutes of Health—NIH, USA) and CellSense v2.3 (Olympus).

UV-microscopy was used to investigate radiation-induced changes in the spatial distribution and relative abundance of chlorophylls and phenolic auto-fluorescent compounds in tissues. Fixed samples were observed using an epifluorescence microscope (Olympus BX53, Olympus Corporation, Japan) equipped with appropriate filter sets.

Chlorophyll autofluorescence was detected using a Cy5 filter set (excitation 620–650 nm, dichroic mirror 660–670 nm, emission 660–700 nm), taking advantage of the natural red fluorescence emitted by chlorophyll pigments. Polyphenol autofluorescence was detected using a DAPI filter set (excitation 340–380 nm, dichroic mirror > 400 nm, emission 420–480 nm), which allows visualization of phenolic compounds due to their blue–green fluorescence emission.

Images were acquired under identical exposure time and illumination settings for all treatments to ensure comparability. For each treatment and time point (12 and 63 days after irradiation), at least five fields of view per sample were captured at the same magnification.

Quantitative image analysis was performed using ImageJ software (NIH, USA). For each image, fluorescence intensity was measured as a mean gray value after background subtraction (Schneider et al. 2012). In addition, the area occupied by fluorescent signal was quantified by applying a consistent threshold to all images and expressed as a percentage of total tissue area. Chlorophyll and polyphenol fluorescence data were analyzed separately.

This approach allowed the detection of dose- and time-dependent changes in pigment distribution and antioxidant-related compounds at the tissue level.

### Pigment quantification, antioxidant activity, and total polyphenols

Photosynthetic pigments were extracted from frozen tissue (0.04 g FW) using ice-cold 100% acetone. Samples were centrifuged, and absorbance of the supernatant was measured at 662, 630, and 470 nm using a UV–VIS spectrophotometer (Cary 100, Agilent Technologies, USA).

Total chlorophylls (a + b) and carotenoids were calculated according to Lichtenthaler (1987) and expressed as mg g⁻^1^ fresh weight.

Antioxidant capacity was determined using the DPPH radical scavenging assay. Methanolic extracts (0.067 mL) were added to 2 mL of 6 × 10⁻^5^ M DPPH solution and incubated at 37 °C for 20 min. Absorbance was measured at 515 nm, and results were expressed as percentage inhibition relative to a Trolox standard.

Total polyphenol content was quantified using the Folin–Ciocalteu method. Fresh tissue (0.20 g FW) was extracted with 80% methanol, centrifuged, and the supernatant reacted with Folin–Ciocalteu reagent and Na₂CO₃. Absorbance was measured at 765 nm, and results were expressed as mg gallic acid equivalents (GAE) g⁻^1^ fresh weight.

### Elemental analysis

At 63 DAI, moss samples were mineralized in 2% HNO₃ and analyzed by inductively coupled plasma–atomic emission spectroscopy (ICP-AES) using an iCAP 6000 spectrometer (Thermo Scientific, USA). Concentrations of macro- and microelements were quantified and expressed as µg g⁻^1^ dry weight.

### Statistical analysis

All statistical analyses were performed using R software (version 4.4.3). All measurements were performed on three independent biological replicates (*n* = 3) per treatment unless otherwise specified. Data normality and homogeneity of variance were assessed using Shapiro–Wilk and Levene’s tests, respectively, prior to ANOVA. Two-way analysis of variance (ANOVA) was used to assess the effects of irradiation dose and days after irradiation (DAI), followed by Tukey’s HSD post hoc test (*p* ≤ 0.05). Multivariate analyses were conducted to explore treatment-induced changes in overall physiological and biochemical profiles. All quantitative variables were standardized (z-score transformation) prior to analysis. Principal component analysis (PCA) was visualized as biplots showing sample scores and variable loadings on the first two principal components.

To quantify multivariate divergence from control conditions, Euclidean distance from the time-matched control centroid was calculated in standardized trait space for each sampling time (DAI), using the full trait set available for each dataset. The control centroid was estimated exclusively from time-matched control samples, and irradiated samples were projected into the same standardized trait space. This approach provides a synthetic measure of overall phenotypic divergence by integrating multiple standardized traits into a common multivariate framework and has been applied in ecological and multivariate studies to assess treatment-induced divergence and recovery in complex biological systems (Van den Brink and Braak 1999; Trygg et al. 2007). Distances were summarized as mean ± SE; control samples were excluded from graphical representations because their distance from the reference centroid is, by definition, minimal.

Data visualization and multivariate analyses were performed using the *ggplot2* and *FactoMineR* packages.

## Results

### O₂/CO₂ dynamics

Continuous monitoring of O_2_/CO_2_ dynamics using the closed-loop sensor-based system revealed a clear dose-dependent modulation of CO₂ uptake and O₂ production in *T. barbieri *(Table 1).

**Table 1:** Effects of acute X-ray irradiation on CO₂ uptake and O₂ production rates derived from continuous O₂ and CO₂ sensor recordings in the aquatic moss *T. barbieri*. Mean values ± SE are reported. Different letters within each sampling time indicate significant differences among irradiation doses according to Tukey’s HSD test (*p* ≤ 0.05). Asterisks indicate the significance of the dose effect (*p* ≤ 0.05*; *p* ≤ 0.01**; *p* ≤ 0.001***; *NS* not significant)

	CO_2_ uptake (μmol h^−1^ gFW^−1^)	O_2_ production (μmol h^−1^ gFW^−1^)
CTRL	2.78 ± 0.01b	0.25 ± 0.01c
1 Gy	6.82 ± 0.04a	0.52 ± 0.02a
10 Gy	2.74 ± 0.039b	0.33 ± 0.05b
30 Gy	2.91 ± 0.54b	0.25 ± 0.02bc
*p*	**	***

CO₂ uptake rates differed significantly among treatments (*p* ≤ 0.05), with samples exposed to 1 Gy showing the highest values, significantly exceeding the control and the other treatments.

O₂ production followed a similar pattern. The highest O₂ evolution rate was recorded at 1 Gy, whereas both 10 Gy and 30 Gy resulted in significantly lower values, approaching those of the non-irradiated control.

### Photosynthetic performance and photosystem II photochemical activity

**Fig. 2:**
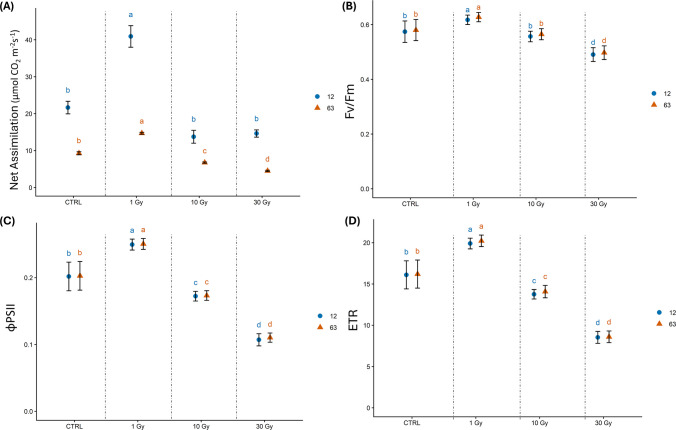
Photosynthetic responses of *T. barbieri* to acute X-ray irradiation. Net assimilation (µmol CO_2_ m^−2^ s·^−1^) (**A**), maximum quantum efficiency of PSII (Fv/Fm) (**B**), effective quantum yield of PSII (ΦPSII) (**C**), and electron transport rate (ETR) (**D**) measured at 12 (blue circle) and 63 (orange triangle) days after irradiation (DAI). Data are shown as mean ± SE. Different letters indicate significant differences among irradiation doses within the same DAI (Tukey’s HSD, *p* ≤ 0.05)

Acute X-ray irradiation significantly affected the photosynthetic performance of *T. barbieri*, inducing clear dose-dependent responses across all analyzed parameters (Fig. 2). Net photosynthetic assimilation was significantly influenced by irradiation dose, sampling time (DAI), and their interaction (Fig. 2A; *p* ≤ 0.001), indicating that the magnitude and direction of the response varied between the two sampling times.

At 12 DAI, net assimilation exhibited a pronounced stimulatory response at 1 Gy, reaching values significantly higher than those observed in the control and at higher doses (10 and 30 Gy). In contrast, at 63 DAI, assimilation rates were overall lower across treatments, although a relative maximum was still observed at 1 Gy, followed by a progressive decline toward the highest irradiation dose. The significant Dose × DAI interaction reflects this temporal modulation of the dose–response relationship, with low-dose stimulation evident primarily at the earlier sampling point.

In contrast to gas exchange measurements, photochemical indices were driven almost exclusively by irradiation dose. For all photochemical parameters, the main effect of dose was highly significant (*p* ≤ 0.001), whereas neither sampling time (DAI) nor the Dose × DAI interaction showed significant effects, indicating that photochemical responses remained stable between 12 and 63 DAI.

The maximum photochemical efficiency of PSII (Fv/Fm; Fig. 2B) reached its highest value at 1 Gy, while control and 10 Gy treatments remained within the typical range of functional PSII. Exposure to 30 Gy resulted in a marked reduction of Fv/Fm, indicative of reduced PSII functionality. Similarly, the effective quantum yield of PSII (ΦPSII; Fig. 2C) and the electron transport rate (ETR; Fig. 2D) showed clear dose-dependent trends, with enhanced values at low irradiation dose (1 Gy) compared with the control, followed by a progressive decline at 10 and 30 Gy.

### Morphological and microscopic responses

**Fig. 3:**
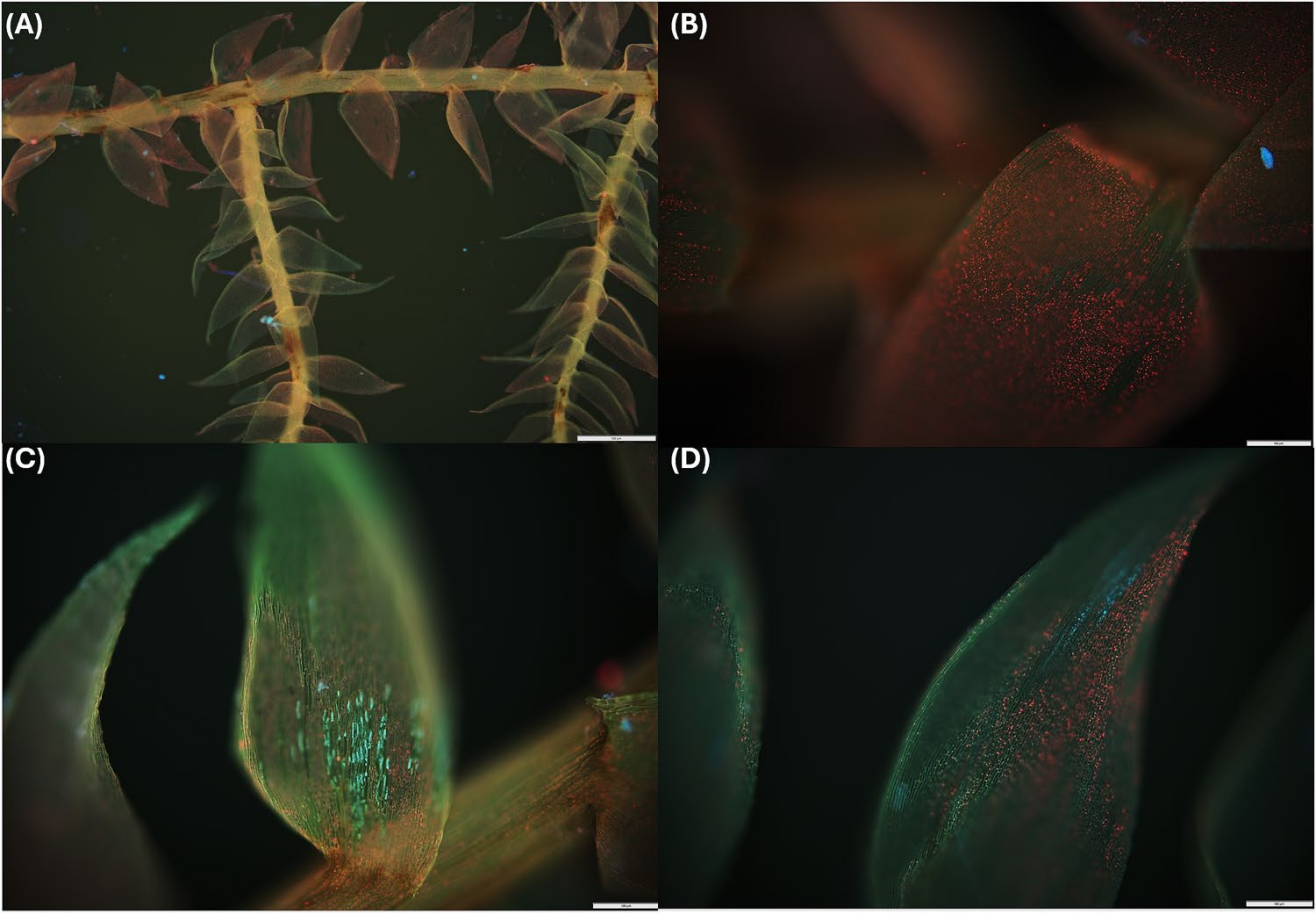
Structural organization and fluorescence-based mapping in the aquatic moss *T. barbieri* under X-ray irradiation. **A** Representative view of shoot architecture and ramification pattern. **B** Chlorophyll autofluorescence (red). **C** Fluorescence associated with phenolic compounds (blue). **D** Composite fluorescence image showing the spatial distribution and partial co-localization of chlorophyll (red) and phenolic compounds (blue) within leaf tissues. Scale bars: 500 µm (**A**), 100 µm (**B**–**D**)

Microscopic observations highlighted clear irradiation-induced modifications in shoot architecture and tissue-level fluorescence patterns of *T. barbieri* (Fig. 3), which were quantitatively confirmed by the morphological and fluorescence-based measurements reported in Tables 2 and 3. Morphological and fluorescence-based traits showed clear dose-dependent responses to acute X-ray irradiation, with distinct patterns between early (12 DAI) and late (63 DAI) sampling times.

Ramification density and length increased in all irradiated treatments compared with the control (*p* ≤ 0.01 and *p* ≤ 0.001, respectively) (Table 2).

Phenolic compounds’ autofluorescence intensity (Table 3) was significantly influenced by dose at 12 DAI (*p* ≤ 0.001), showing a marked increase at 1 Gy relative to the control, followed by a progressive decline at higher doses. In contrast, the area occupied by phenolic fluorescence was higher in controls and significantly decreased with increasing dose (*p* ≤ 0.001).

At 63 DAI, fluorescence-based parameters continued to exhibit significant dose-dependent responses. Phenolic autofluorescence intensity remained significantly affected by dose (*p* ≤ 0.001), again peaking at 1 Gy and decreasing at higher doses. A similar pattern was observed for the phenolics’ fluorescent area, which was significantly reduced at 10 and 30 Gy compared with the control and 1 Gy treatments (*p* ≤ 0.001).

Chlorophyll autofluorescence intensity (Table 3) was also significantly influenced by irradiation dose at both 12 and 63 DAI (*p* ≤ 0.01), with the highest values recorded at 1 Gy and a pronounced reduction at 30 Gy. Conversely, the chlorophylls’ auto-fluorescent area did not show significant differences among treatments (NS), indicating that irradiation mainly altered pigment intensity rather than its spatial distribution.

**Table 2:** Effects of acute X-ray irradiation on morphological traits in the aquatic moss *T. barbieri*. Ramification density (branches mm⁻^2^) and ramification length (mm) are shown. Mean values ± SE are reported. Different letters within each sampling time indicate significant differences among irradiation doses according to Tukey’s HSD test (*p* ≤ 0.05). Asterisks indicate the significance of the dose effect (*p* ≤ 0.05*; *p* ≤ 0.01**; *p* ≤ 0.001***; *NS* not significant)

	Ramification density (branches mm^−2^)	Ramification length (mm)
CTRL	0.027 ± 0.014b	1.039 ± 0.527b
1 Gy	0.128 ± 0.019a	2.169 ± 0.124a
10 Gy	0.114 ± 0.022a	2.257 ± 0.207a
30 Gy	0.134 ± 0.016a	2.656 ± 0.196a
*p*	**	***

**Table 3:** Effects of acute X-ray irradiation (1, 10, 30 Gy plus a non-irradiated control, CTRL) on fluorescence-based indicators in the aquatic moss *T. barbieri*. Phenolic compounds autofluorescence intensity and area (%), and chlorophyll autofluorescence intensity and area (%) quantified at 12 and 63. Mean values ± SE are reported. Different letters within each sampling time indicate significant differences among irradiation doses according to Tukey’s HSD test (*p* ≤ 0.05). Asterisks indicate the significance of the dose effect (*p* ≤ 0.01**; *p* ≤ 0.001***; *NS* not significant)

		Phenolic compounds autofluorescence intensity	Phenolic compounds autofluorescent area (%)	Chlorophyll autofluorescence intensity	Chlorophyll autofluorescent area (%)
12 DAI	CTRL	88.45 ± 0.14b	2.09 ± 0.42a	125.16 ± 2.04b	34.49 ± 3.52a
	1 Gy	99.72 ± 2.84a	0.97 ± 0.39b	138.87 ± 8.49a	37.84 ± 3.24a
	10 Gy	95.13 ± 2.46a	0.49 ± 0.14c	118.85 ± 4.54b	37.49 ± 3.47a
	30 Gy	85.21 ± 2.91b	0.41 ± 0.14c	98.73 ± 4.97c	30.80 ± 3.39a
*p*		***	***	**	*NS*
63 DAI	CTRL	79.61 ± 0.64c	1.99 ± 0.40a	112.64 ± 10.8b	33.46 ± 3.41a
	1 Gy	89.75 ± 2.56a	0.95 ± 0.38b	124.98 ± 7.64a	36.71 ± 3.15a
	10 Gy	85.62 ± 2.21b	0.47 ± 0.14c	106.96 ± 4.08b	36.37 ± 3.36a
	30 Gy	76.68 ± 2.62d	0.40 ± 0.14c	88.86 ± 4.48c	29.87 ± 2.32a
*p*		***	***	**	*NS*

### Pigment content and antioxidant responses

**Fig. 4:**
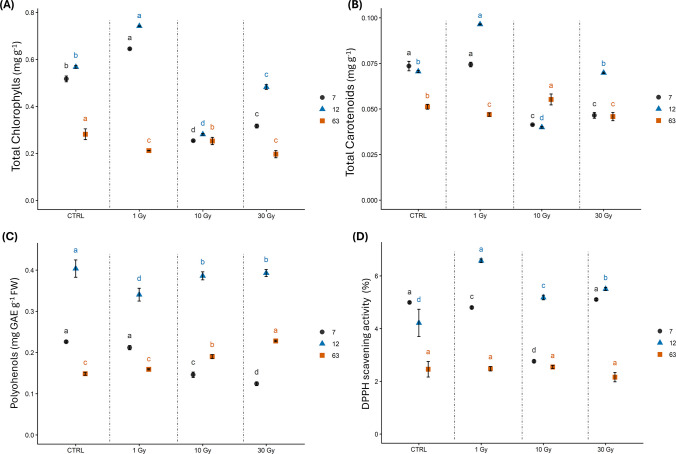
Dose- and time-dependent modulation of photosynthetic pigments and antioxidant-related traits in *T. barbieri*. Total chlorophyll content (**A**), total carotenoid content (**B**), total polyphenol concentration (**C**), and DPPH radical scavenging activity measured at 7 (black circle), 12 (blue triangle), and 63 (orange square) days after irradiation (DAI) (**D**). Data are shown as mean ± SE. Different letters indicate significant differences among irradiation doses within the same DAI (Tukey’s HSD, *p* ≤ 0.05)

Consistent with the structural and physiological patterns observed, irradiation dose and sampling time significantly modulated pigment content and antioxidant-related traits in *T. barbieri* (Fig. 4).

Total chlorophyll content (Fig. 4A) was strongly influenced by irradiation dose, days after irradiation (DAI), and their interaction (*p* ≤ 0.001 for all factors). Across sampling times, chlorophyll levels were consistently stimulated at 1 Gy compared with the control, whereas higher doses induced a marked reduction. However, at 63 DAI, values at the dose of 1 Gy decreased sharply. Temporal dynamics further modulated this response, with the highest chlorophyll content recorded at 12 DAI and a general decline at 63 DAI.

A similar pattern was observed for total carotenoids (Fig. 4B). Carotenoid content was significantly influenced by dose, DAI, and their interaction (*p* ≤ 0.001), with 1 Gy consistently promoting higher carotenoid accumulation relative to CTRL and higher doses, at all sampling times except for 63 DAI. In contrast, 10 and 30 Gy resulted in pronounced carotenoid depletion, especially at later sampling times, suggesting impaired photoprotective capacity under sustained high-dose exposure.

Polyphenol content (Fig. 4C) showed a strong temporal component, with DAI and the Dose × DAI interaction being highly significant (*p* ≤ 0.001), while the main effect of dose alone was weaker (*p* ≤ 0.05). At 7 and 12 DAI, polyphenol levels were generally elevated at low to moderate doses, whereas at 63 DAI a clear dose-dependent increase emerged, with the highest value observed at 30 Gy.

In line with polyphenol dynamics, DPPH radical scavenging activity (Fig. 4D) was significantly affected by dose, DAI, and their interaction (*p* ≤ 0.001 for all factors). Antioxidant capacity peaked at early sampling times under low irradiation (1 Gy), while higher doses induced variable responses depending on time. At 63 DAI, DPPH activity converged across treatments, indicating a stabilization of antioxidant potential during the recovery phase despite persistent differences in pigment composition.

Concerning the elemental analysis, no difference was detected among treatments (please see supplementary information).

### Integrated dose–time response patterns

**Fig. 5:**
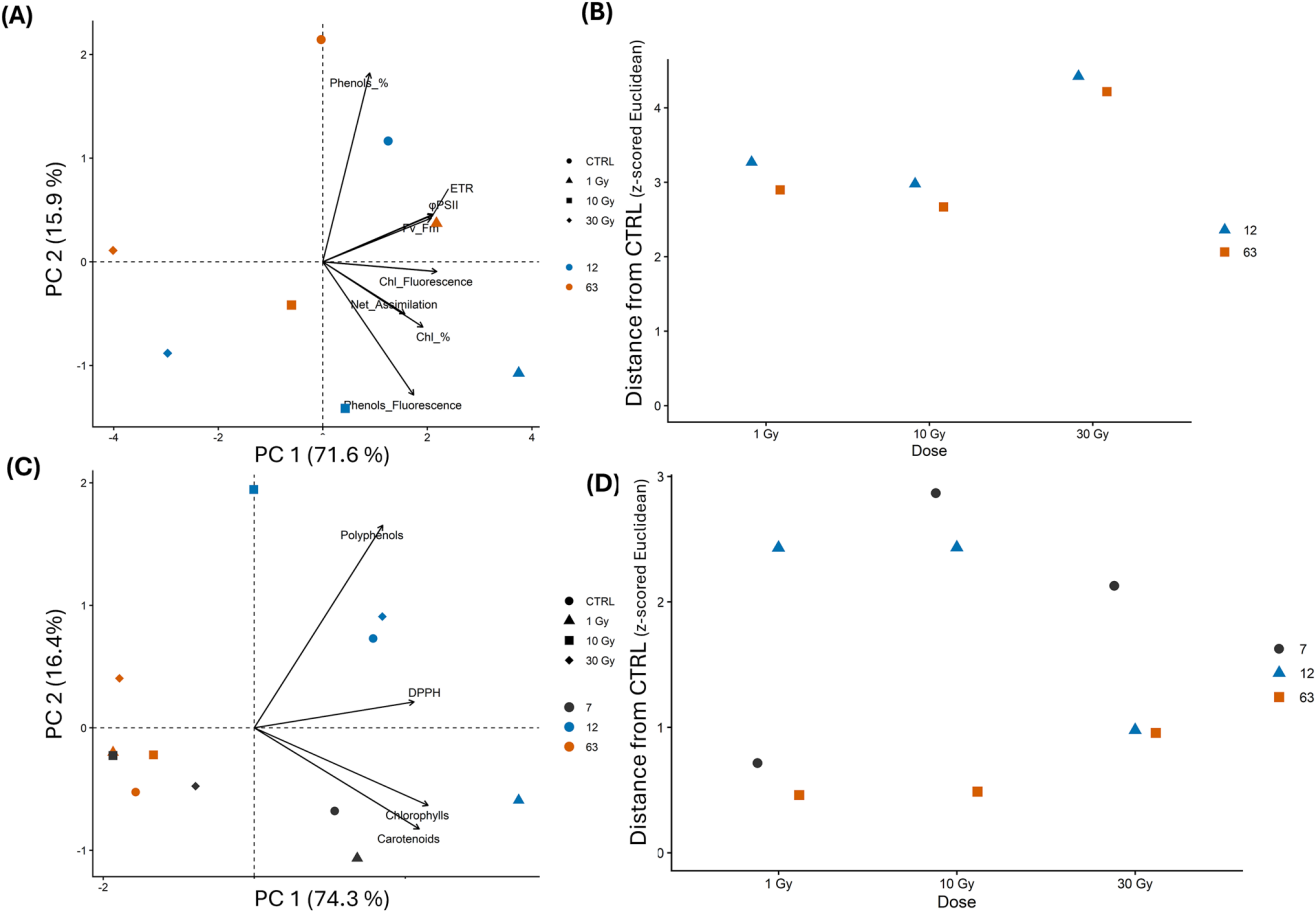
Multivariate analysis of physiological and biochemical responses to X-ray irradiation over time. Principal component analysis (PCA) biplots and corresponding multivariate distance-from-control analyses are shown for the physiological dataset (**A**–**B**) and the biochemical dataset (**C**–**D**). Symbols indicate irradiation dose (CTRL, 1 Gy, 10 Gy, 30 Gy), while colors represent sampling time (days after irradiation, DAI)

Principal component analysis (PCA) revealed clear dose- and time-dependent shifts in the multivariate physiological profiles (Fig. 5). In the first PCA (Fig. 5A) of the physiological data, the first two principal components explained 71.6% (PC1) and 15.9% (PC2) of the total variance. PC1 primarily captured variation associated with photosynthetic and fluorescence-related traits (ETR, ΦPSII, Fv/Fm, net assimilation, and chlorophyll-related variables), whereas PC2 was mainly driven by phenolic-related variables. Samples exposed to irradiation were clearly separated from their respective controls along PC1 at early sampling times, indicating a marked treatment-induced divergence of the physiological profile.

Consistent with the PCA patterns, the multivariate distance-from-control analysis for this dataset (Fig. 5B) showed elevated distances at 12 DAI across all doses, indicating a strong deviation from the control phenotype shortly after irradiation. In contrast, distances were consistently lower at 63 DAI.

A similar pattern emerged in the second PCA (Fig. 5C) about the biochemical data, where the first two principal components explained 74.3% (PC1) and 16.4% (PC2) of the total variance. PC1 was largely associated with pigment-related traits (chlorophylls and carotenoids), while PC2 was mainly influenced by antioxidant-related variables (polyphenols and DPPH). As observed before, irradiated samples at early sampling times were more dispersed relative to controls, whereas later sampling times showed increased clustering and reduced separation in the multivariate space.

The distance-from-control analysis relative to this dataset (Fig. 5D) further supported this temporal trend. Distances from the control centroid were highest at early sampling times and decreased markedly at 63 DAI across all irradiation doses.

## Discussion

### Dose-dependent responses and hormesis in an aquatic bryophyte

The present study demonstrates that acute X-ray irradiation induces clear dose-dependent responses in the aquatic moss *T. barbieri*, characterized by stimulation at low dose and progressive functional reduction at higher doses, confirming our first hypothesis. Overall, low-dose exposure (1 Gy) enhanced photosynthetic performance, PSII photochemical efficiency, pigment content, and antioxidant-related traits, whereas higher doses (10–30 Gy) reduced physiological functioning. This biphasic dose–response pattern is consistent with a hormesis-like response a widely recognized adaptive phenomenon in organisms exposed to low-intensity environmental stressors, including radiation-induced oxidative stress, which activates compensatory and protective mechanisms rather than causing net damage (Calabrese and Baldwin 2003; Calabrese and Mattson 2017), and suggests the activation of adaptive mechanisms at low irradiation levels. While hormetic responses to ionizing radiation have been widely documented in vascular plants and microalgae, evidence in aquatic bryophytes remains limited. For example, in *Brassica rapa* L. microgreens X-ray exposure significantly enhanced biomass accumulation and hypocotyl elongation at 1 Gy (De Francesco et al. 2025). Comparable low-dose stimulatory effects in higher plants have been reported in *Phoenix dactylifera* L. seedlings (Al-Enezi et al. 2012). Nevertheless, because only three discrete dose levels were tested here, the present study does not allow a precise characterization of the hormetic zone or the identification of exact biological thresholds. Future studies including intermediate doses would be valuable for refining dose–response models and quantifying transition points between stimulation and inhibition. In addition, limited beam-time availability reinforced the choice of a restricted number of biologically informative dose levels while maintaining analytical consistency across all investigated endpoints.

Our results extend this framework to a non-vascular aquatic primary producer, indicating that *T. barbieri* can perceive low-intensity ionizing radiation as an environmental signaling cue, activating compensatory physiological and metabolic responses rather than inducing net functional damage.

The parallel stimulation of gas exchanges, PSII efficiency, and electron transport rate at low dose is consistent with the activation of redox-regulated adaptive responses that may act as a regulatory signal for up-regulating photosynthetic and metabolic processes. However, because ROS production and oxidative damage markers were not directly quantified, the involvement of specific ROS-mediated signaling pathways remains inferential and should be interpreted with caution.

Low-dose stimulatory responses have been previously reported in higher plants exposed to environmentally relevant ionizing radiation, where redox-mediated signaling modulates photosynthetic performance and antioxidant metabolism (Horneck et al. 2010; Azzam et al. 2012). Overall, the absence of temporal effects on photochemical parameters suggests that PSII functionality remained relatively stable during the post-irradiation recovery period. In contrast, net carbon assimilation exhibited a stronger sensitivity to both irradiation dose and time, indicating that carbon metabolism was more responsive to prolonged oxidative constraints than photochemical efficiency per se. In *T. barbieri*, the simultaneous increase in polyphenol content and antioxidant activity further supports the idea that low-dose irradiation triggers an adaptive metabolic reprogramming aimed at enhancing stress tolerance. The identification of a stimulatory threshold at 1 Gy and functional impairment above 10 Gy may contribute to defining biologically informed dose–response models for freshwater primary producers. Importantly, transient functional recovery observed at intermediate doses highlights the need to incorporate resilience and physiological recovery into risk characterization, rather than relying solely on acute toxicity thresholds.

### Temporal dynamics, antioxidant buffering, and functional resilience

Beyond dose effects, the temporal evolution of responses emerged as a critical component of the moss reaction to irradiation. At early sampling times, irradiated mosses showed pronounced deviations from control conditions across multiple physiological and biochemical traits, reflecting the immediate impact of acute oxidative stress. However, 63 days after irradiation, multivariate analyses revealed a consistent reduction in distance from control profiles across all doses. This pattern, quantitatively captured by the distance-from-control analysis, indicates a progressive convergence toward physiological states resembling those of non-irradiated controls. This analytical approach detects system-level functional shifts, providing an integrative assessment of stress impact and recovery that is particularly relevant for complex environmental disturbances (Van den Brink and Braak 1999; Trygg et al. 2007). Because the metric integrates multiple correlated traits into a single descriptor of phenotypic divergence, it is particularly useful for identifying broad patterns of stress response and recovery that may not be evident from individual variables alone. However, the relatively low number of biological replicates limits the statistical power of multivariate analyses and may reduce the stability of sample ordination patterns. Therefore, PCA and distance-from-control analyses should be interpreted primarily as exploratory and integrative tools supporting the evaluation of overall response trajectories rather than as definitive inferential evidence.

Importantly, this convergence does not imply complete recovery of all measured traits. Several physiological variables, including net assimilation, PSII photochemical efficiency, electron transport rate, and antioxidant activity, showed a clear tendency toward recovery at later sampling times, contributing substantially to the observed reduction in multivariate divergence from control samples. In contrast, some biochemical and structural traits, including carotenoid content, phenolic fluorescence distribution, chlorophyll fluorescence intensity at the highest dose, and shoot architectural parameters, remained altered relative to controls. Therefore, the observed convergence should be interpreted as partial physiological recovery and functional resilience rather than complete restoration of the pre-disturbance phenotype (Holling 1973; Gunderson 2000; Mittler 2017). Although direct ROS quantification and oxidative damage measurements were not performed, the coordinated modulation of antioxidant capacity, phenolic accumulation, and photochemical parameters is consistent with a redox-mediated response. Nevertheless, the underlying mechanisms cannot be conclusively demonstrated based on the present dataset. Indeed, antioxidant dynamics appear to play a central role in this process. Early increases in polyphenol content and scavenging activity likely contributed to buffering oxidative stress, while the subsequent stabilization of antioxidant levels at later stages suggests a re-establishment of redox homeostasis. Similar temporal trajectories have been reported in plants exposed to acute abiotic stress, where initial antioxidant activation is followed by functional stabilization, once the stress intensity declines (Mittler 2017; Einor et al. 2016). Importantly, this convergence does not imply that all traits returned to control values. Some pigment-related and structural parameters remained altered even at later stages. Rather, it indicates functional resilience, meaning that the moss progressively re-established an integrated physiological balance despite the persistence of specific biochemical or structural modifications.

### Structural plasticity and ecological relevance

Beyond physiological and biochemical responses, acute irradiation induced marked changes in shoot architecture and tissue-level fluorescence patterns, highlighting the role of structural plasticity in stress tolerance. Increased ramification density in irradiated samples indicates a reorganization of growth patterns, potentially enhancing surface area for gas exchange and light capture. Such structural reorganization may enhance light interception and gas exchange efficiency, representing an adaptive trait under environmental stress conditions commonly encountered in polluted or radiation-impacted freshwater habitats.

Structural remodeling is a well-recognized adaptive strategy in bryophytes, allowing them to cope with fluctuating environmental conditions such as desiccation, nutrient limitation, and pollution (Proctor et al. 2007). In the present study, enhanced ramification under irradiation may represent a compensatory growth strategy, partially offsetting functional losses at higher doses and contributing to overall system stability.

While the chlorophyll auto-fluorescent area remained relatively stable, fluorescence intensity showed a clear dose-dependent modulation, suggesting that irradiation primarily influenced pigment concentration and organization rather than causing extensive pigment disruption. This modulation may also reflect a partial reduction in chlorophyll biosynthesis, in addition to structural rearrangements within the photosynthetic apparatus. Similar patterns have been reported in radiation-exposed plants, where alterations in pigment content and photochemical properties occur without overt structural degradation of tissues (Amitrano et al. 2026). The absence of significant changes in elemental composition further indicates that radiation-induced effects were primarily related to oxidative and physiological processes rather than to nutrient imbalance.

The ability of *T. barbieri* to tolerate acute ionizing radiation and to progressively restore integrated physiological functioning has important ecological and applied implications. Aquatic bryophytes are widely used as bioindicators and biofilters in freshwater ecosystems due to their high accumulation capacity and sensitivity to environmental stressors. Our findings suggest that, beyond passive contaminant accumulation, aquatic mosses can maintain physiological stability even under acute dose exposure and concurrent oxidative stress, supporting the maintenance of physiological performance under acute and localized oxidative disturbances. From an ecological risk assessment perspective, the observed dose-dependent response patterns provide useful organism-level information that may support the integration of eco-physiological endpoints into well-documented weight-of-evidence frameworks. Aquatic bryophytes, due to their high surface-to-volume ratio and rapid physiological responsiveness, may represent suitable early-warning bioindicator species in freshwater systems exposed to episodic radiation inputs. It should be noted that the higher doses investigated in this study are not intended to reproduce typical environmental exposure conditions. Rather, they were included to define the response envelope of the species under severe oxidative stress and to identify physiological thresholds that may support ecological risk assessment frameworks. Such threshold-oriented approaches are commonly employed in radiobiology to complement environmentally realistic exposure studies. An important limitation of the present study is the absence of direct measurements of reactive oxygen species (ROS) and oxidative damage markers, such as hydrogen peroxide accumulation, lipid peroxidation, or membrane integrity indicators. Consequently, mechanistic interpretations linking irradiation effects to oxidative stress responses are based on indirect evidence derived from antioxidant activity, phenolic metabolism, and physiological performance. Future studies combining eco-physiological measurements with direct biochemical assessment of oxidative stress will be necessary to confirm the mechanisms proposed here.

## Conclusions

This study demonstrates that the aquatic moss *T. barbieri* exhibits dose-dependent physiological and biochemical responses to acute X-ray irradiation, characterized by low-dose stimulation and progressive functional impairment at higher doses. The integration of univariate and multivariate analyses revealed a time-dependent reduction in divergence from control conditions, indicating a capacity for physiological recovery following irradiation (Fig. 5). Although several physiological traits showed substantial recovery over time, some biochemical and structural parameters remained altered at the final sampling point, indicating that resilience was only partial and trait-dependent.

The combination of low-dose stimulatory responses consistent with hormesis, antioxidant buffering, and multivariate convergence highlights the potential of aquatic bryophytes as model organisms for studying stress resilience in extreme environments but also as highly resilient primary producers. In fact, despite the difficult comparison with higher plants in the absence of comparative experimental data, the observed response patterns are consistent with the well-documented stress tolerance of bryophytes, which have evolved a range of physiological and structural mechanisms enabling survival under fluctuating and adverse environmental conditions (Proctor et al. 2007). This is particularly relevant for ecosystems exposed to multiple interacting stressors, as well as for space-related and planetary analogue research, where biological systems are subjected to elevated radiation levels. Overall, the present study places aquatic mosses as resilient biological components capable of sustaining functional performance under acute radiation stress, reinforcing their value in environmental monitoring and in experimental frameworks aimed at defining tolerance limits of aquatic primary producers. However, because ROS and oxidative damage markers were not directly quantified, the proposed oxidative stress mechanisms should be regarded as hypotheses supported by indirect physiological and biochemical evidence rather than definitive mechanistic demonstrations.

## Supplementary Information

Below is the link to the electronic supplementary material.

ESM 1 (DOCX 18.6 KB)

## Data Availability

Data will be made available after reasonable request to the corresponding author.
