# Supplementary material for: Dose-dependent stress responses of the aquatic moss Taxiphyllum barbieri under acute X-ray exposure: implications for environmental risk assessment
Source: Environ Sci Pollut Res Int. 2026 Jul 16;33(23):11915–29. doi: 10.1007/s11356-026-38065-4 (PMC13424475; doi:10.1007/s11356-026-38065-4)
Supplement: Supplementary file 1 — (DOCX 18.6 KB) [file 11356_2026_38065_MOESM1_ESM.docx]

**ELECTRONIC SUPPLEMENTARY INFORMATIONS**

**Title:** Dose-dependent stress responses of the aquatic moss *Taxiphyllum barbieri* under acute X-ray exposure: implications for environmental risk assessment.

**Authors**: Chiara Amitrano^1^*, Veronica De Micco^1^, Gian Pietro Di Sansebastiano^2^, Mariagabriella Pugliese^3^, Cecilia Arrichiello^4^, Paolo Muto^4^, Stefania De Pascale^1^, Carmen Arena^5^

^1^Department of Agricultural Sciences, University of Naples Federico II, Portici (Naples), ITALY
^2^Department of Biological and Environmental Sciences and Technologies (Di.S.Te.B.A.),University of Salento, Lecce, ITALY.
^3^Department of Physics Ettore Pancini, University of Naples Federico II, Naples, ITALY.
^4^Radiotherapy Unit, Istituto Nazionale Tumori—IRCCS—Fondazione G. Pascale, Naples, ITALY.
^5^Department of Biology, University of Naples Federico II, Naples, ITALY.

*corresponding author: [chiara.amitrano@unina.it](mailto:chiara.amitrano@unina.it), +390812539856

Elemental composition (mean ± standard error) of *Taxiphyllum barbieri* after exposure to increasing X-ray doses. Values represent mean ± standard error (n = 3). Differences among treatments were not statistically significant (p > 0.05).

| **Element** | **CTRL** | **1 Gy** | **10 Gy** | **30 Gy** |
| --- | --- | --- | --- | --- |
| **Ca** | 57079.34 ± 7562.45a | 70178.18 ± 14194.25a | 55331.41 ± 15181.73a | 65581.60 ± 31287.87a |
| **Cd** | 0.1660 ± 0.0134a | 0.1619 ± 0.0181a | 0.1894 ± 0.0193a | 0.1746 ± 0.0153a |
| **Cu** | 19.76 ± 9.11a | 21.24 ± 8.02a | 16.40 ± 2.88a | 19.39 ± 3.11a |
| **Mn** | 3.53 ± 1.61a | 7.45 ± 2.44a | 4.63 ± 1.17a | 3.74 ± 1.85a |
| **Fe** | 28.63 ± 4.89a | 49.00 ± 7.36a | 31.68 ± 2.45a | 28.79 ± 16.81a |
| **Cr** | 0.92 ± 0.12a | 0.94 ± 0.36a | 0.74 ± 0.17a | 1.16 ± 0.67a |
| **Ni** | 6.77 ± 3.79a | 6.66 ± 2.26a | 4.92 ± 1.25a | 4.82 ± 3.36a |
| **Al** | 54.68 ± 10.35a | 66.33 ± 17.70a | 53.32 ± 10.80a | 70.16 ± 40.51a |
| **K** | 4715.10 ± 548.47a | 4790.11 ± 755.52a | 5119.68 ± 220.52a | 4565.99 ± 2636.15a |
| **Na** | 409.76 ± 190.59a | 384.23 ± 155.74a | 541.79 ± 148.78a | 340.61 ± 196.65a |
| **Mg** | 3963.49 ± 584.91a | 4141.78 ± 155.74a | 4612.92 ± 785.31a | 4244.83 ± 2450.76a |
| **Ba** | 20.17 ± 3.72a | 52.96 ± 12.96a | 31.91 ± 6.95a | 30.57 ± 17.65a |
| **Sr** | 104.91 ± 5.59a | 123.37 ± 23.37a | 107.52 ± 13.44a | 107.52 ± 85.89a |
| **B** | 54.18 ± 7.36a | 58.35 ± 12.37a | 45.60 ± 9.39a | 48.75 ± 26.22a |
| **Li** | 0.80 ± 0.01a | 1.30 ± 0.85a | 1.14 ± 0.40a | 1.05 ± 0.67a |
| **Se** | 0.59 ± 0.17a | 0.19 ± 0.16a | 0.49 ± 0.07a | 0.82 ± 0.18a |
| **As** | 0.23 ± 0.01a | 0.19 ± 0.16a | 0.07 ± 0.07a | 0.35 ± 0.18a |
| **Zn** | 49.92 ± 6.15a | 48.10 ± 48.10a | 46.64 ± 46.64a | 55.60 ± 32.10a |
